# Changing Antiarrhythmic Drug Regimen in Patients with Amiodarone and Ablation Refractory Ventricular Tachyarrhythmias Is Associated with Increased Implantable Cardioverter Defibrillator Shocks—A Retrospective Analysis from a Large Tertiary Center

**DOI:** 10.3390/jcm14092859

**Published:** 2025-04-22

**Authors:** Florian Doldi, Kevin Willy, Julian Wolfes, Christian Ellermann, Steffen Taeger, Felix K. Wegner, Fatih Güner, Dennis Korthals, Benjamin Rath, Gerrit Frommeyer, Julia Köbe, Florian Reinke, Philipp Sebastian Lange, Lars Eckardt

**Affiliations:** Department for Cardiology II: Electrophysiology, University Hospital Munster, 48149 Munster, Germany; kevin.willy@ukmuenster.de (K.W.); julian.wolfes@ukmuenster.de (J.W.); staeger@uni-muenster.de (S.T.); felix.wegner@ukmuenster.de (F.K.W.); fatih.guener@ukmuenster.de (F.G.); dennis.korthals@ukmuenster.de (D.K.); benjamin.rath@ukmuenster.de (B.R.); gerrit.frommeyer@ukmuenster.de (G.F.); julia.koebe@ukmuenster.de (J.K.); florian.reinke@ukmuenster.de (F.R.); philippsebastian.lange@ukmuenster.de (P.S.L.); lars.eckardt@ukmuenster.de (L.E.)

**Keywords:** implantable cardioverter defibrillator, ICD shocks, antiarrhythmic drugs, amiodarone, flecainide

## Abstract

**Background and Objective:** Implantable Cardioverter Defibrillators (ICDs) are crucial in treating ventricular tachyarrhythmias (VTs) and preventing sudden cardiac death. However, ICD shocks are linked to higher mortality and a lower quality of life. Many patients suffer from recurrent VTs despite concomitant antiarrhythmic drug (AAD) therapy with amiodarone, and it is unclear if changing the AAD while on chronic amiodarone therapy is beneficial. Hence, we investigated the impact of changing the AAD on the incidence of appropriate ICD shocks in patients on chronic amiodarone, impaired LV function, and at least one previous VT ablation. **Methods and Results:** We retrospectively analyzed 131 ICD patients (LVEF < 40%) from a single-center registry. All were on chronic amiodarone and had undergone VT ablation. The mean age was 66.0 ± 12.8 years; 82.4% were male; and the follow-up period averaged 5.8 ± 0.6 years. Ischemic cardiomyopathy was present in 52.7% of patients. AAD therapy was changed in 49 patients (37.4%), primarily due to inefficacy (40.8%), intolerance (16.3%), or other reasons (42.9%). Of those, 8 received flecainide (≥200 mg) and 41 sotalol (≥240 mg); 82 (62.6%) continued amiodarone. VT re-ablation was performed in 23.7%. During follow-up, 11 patients (8.4%) died and 18 (13.7%) received appropriate ICD shocks—17 with changed AAD vs. 1 with continued amiodarone (*p* ≤ 0.01). A multivariate regression showed that switching from amiodarone to flecainide or sotalol was significantly associated with increased ICD shock risk (OR 34.9; 95% CI 4.3–283.8; *p* < 0.01). **Conclusions:** In patients on chronic amiodarone with severely impaired LV function and at least one previous VT ablation, changing AAD therapy to flecainide or sotalol is associated with an increased incidence of appropriate ICD shocks.

## 1. Introduction

For many years, implantable cardioverter defibrillators (ICDs) have served as a cornerstone in the prevention of sudden cardiac death (SCD) [1]. Despite significant advances in heart failure management, recent randomized trials [2,3,4,5,6] have reported remaining high SCD rates highlighting the persistent relevance of ICD therapy among high-risk patients [7]. The observed benefits of guideline-directed medical therapy (GMDT) have primarily translated into a reduction in all-cause mortality with limited data on the effect on sudden cardiac death [8].

Most patients with an ICD suffer from heart failure with reduced left ventricular ejection fraction (LVEF) due to an ischemic or non-ischemic cardiomyopathy [9,10,11]. Apart from the effective termination of ventricular tachycardias (VTs) by anti-tachycardic pacing (ATP) and/or shocks, ICD therapy depends on the correct discrimination of VTs from supraventricular tachycardias (SVTs) [12]. Current data show an association of increased mortality and decreased quality of life after shock delivery [13]. Therefore, an overall reduction in ICD shocks is an important therapeutic target. In the Optimal Pharmacological Therapy in Cardioverter Defibrillator (OPTIC) study [14,15] amiodarone in conjunction with a beta-blocker significantly reduced the risk of ICD shocks. However, the side effects of amiodarone therapy are relevant and often refrain patients from continuing therapy [16,17]. Therefore, some patients undergo changes in their antiarrhythmic drug (AAD) regimen either for ineffectiveness or intolerance of amiodarone. However, the impact on the outcome due to changes in antiarrhythmic regimen in these patients is unclear [18,19]. Hence, we sought to investigate the influence of changes in the AAD regimen on the incidence of appropriate ICD shocks in patients with ischemic/non-ischemic cardiomyopathy who had undergone at least one catheter ablation for VT and presented with amiodarone refractory VT.

## 2. Pharmacological Aspects

Amiodarone [20], sotalol [21], and flecainide [22] are antiarrhythmic drugs with distinct mechanisms targeting various ionic channels in the heart. Amiodarone, a class III antiarrhythmic, primarily inhibits potassium rectifier currents, leading to prolonged action potential duration and effective refractory period [23]. This reduces myocyte excitability, thereby preventing reentrant arrhythmias and ectopic foci. Unlike other class III agents, amiodarone also blocks sodium and calcium channels and beta-adrenergic receptors, offering a broad spectrum of action but also increasing the risk of side effects like bradycardia and hypotension as well as thyroid dysfunction often leading to the necessity of amiodarone discontinuation. Sotalol, another more selective class III agent, combines β-blocker activity with potassium channel-blocking properties [24]. It prolongs the action potential duration and refractory period across various cardiac tissues. Sotalol’s reverse use-dependent effect increases the risk of QT prolongation and torsades de pointes, particularly in bradycardia. Flecainide, a class IC antiarrhythmic, blocks fast inward sodium channels, particularly during depolarization, prolonging the refractory period [25]. It also inhibits IKr channels, delaying repolarization and reducing reentrant arrhythmias [26]. Flecainide’s action on the His-Purkinje system and its ability to block ryanodine receptors, thereby reducing calcium release and triggered activity, make it particularly useful in treating catecholaminergic polymorphic ventricular tachycardia (CPVT) [27].

## 3. Methods

We retrospectively analyzed a total of 131 consecutive patients >18 years presenting to our outpatient ICD clinic (from 2005 until 2023) who had an implanted ICD, a reduced LVEF (<40%), chronic amiodarone therapy, and at least one previous catheter ablation for VT therapy. Patients with HFpEF, inappropriate shocks, no prior VT ablation, only short-term or discontinued prior amiodarone therapy, as well as not consistently documented changes in antiarrhythmic or ICD therapy, were excluded. Next to antiarrhythmic therapy, all patients received adequate heart failure treatment according to current guidelines. Patients were screened individually for changes in their antiarrhythmic regimen after ICD implantation, changes in the number and type of appropriate ICD therapies delivered, and repeated VT ablations during follow-up. Follow-ups were also performed in our outpatient clinic in 6-month intervals. As these cohorts were retrospectively analyzed, the follow-up duration differed. To address this issue, we added an additional calculation with a uniform follow-up period across all cohorts (2.9 years).

Patients on stable amiodarone therapy with 200 mg daily (longer than 6 months after an initial loading dose of at least 600 mg for 10 days) were compared to those who underwent a change in their AAD regimen to either flecainide (target dosage ≥ 200 mg daily), or sotalol (target dosage > 240 mg). Treatment ineffectiveness was defined as recurring ICD interventions, despite adequate long-term treatment. The primary endpoint of this study was the incidence of ICD shocks after maintaining amiodarone therapy or changing it to either sotalol or flecainide.

## 4. Statistical Analysis

For descriptive statistics, continuous data were presented as means with standard deviation (SD) and medians with interquartile ranges (IQR), respectively. Categorical data were presented as proportions in percentages. The normality of data distribution was assessed using the Shapiro–Wilk test. Comparisons between groups were performed using Pearson’s chi-square test for categorical variables, and Student’s *t*-test or an ANOVA-test for unpaired continuous variables. The risk of ICD shocks was determined using binomial logistic regression and expressed as the Odds Ratio (OR) with a confidence interval (CI) and *p*-value. The cumulative risk of suffering an ICD shock during follow-up was estimated and graphically displayed using Kaplan–Meier curves.

The statistical tests applied yielded two-sided *p*-values with a level (alpha) of <0.05 to determine statistical significance. All analyses were performed with the SPSS software package (SPSS Statistics, Chicago, IL, USA), version 25.0 (2017).

## 5. Results

Among the 131 patients analyzed, we identified 49 patients (37.4%) who underwent a change in antiarrhythmic drug regimen, whereas the remaining *n* = 82 (62.6%) patients continued amiodarone therapy with a daily dose of 200 mg during a mean follow-up (FU) of 5.8 ± 0.6 years. Patients were mostly male (*n* = 108, 82.4%) with a mean BMI of 27.9 ± 5.4 kg/m^2^ and had a mean LVEF of 27.7 ± 6.9% due to either an ischemic (*n* = 69; 52.7%) or non-ischemic (*n* = 62; 47.3%) cardiomyopathy. Patients with changed AAD therapy mostly had a non-ischemic cardiomyopathy (*n* = 26; 53.1 %) (Table 1). A total of 11 patients (8.4%) died during follow-up (*n* = 4, 4.9% under continued amiodarone; *n* = 7, 14.3% after changed AAD therapy). Overall, 18 patients (13.7%) experienced an ICD shock during follow-up. Using a uniform follow-up period across all patient cohorts of 2.9 years, only 10 patients (*n* = 10 under continued amiodarone and *n* = 0 after changed AAD therapy) experienced an appropriate ICD shock. A device upgrade to either a dual-chamber (DDD) or CRT defibrillator was performed in 43 (32.8%) patients.

A change in AAD therapy was performed because of amiodarone ineffectiveness (*n* = 20; 40.8%) or intolerance (*n* = 8; 16.3%) (Table 1 and Table 2). AAD therapy was mainly changed to sotalol with >240 mg in 41 patients (83.7%). Eight patients (16.3%) received ≥200 mg of flecainide instead of amiodarone.

**Figure 1:**
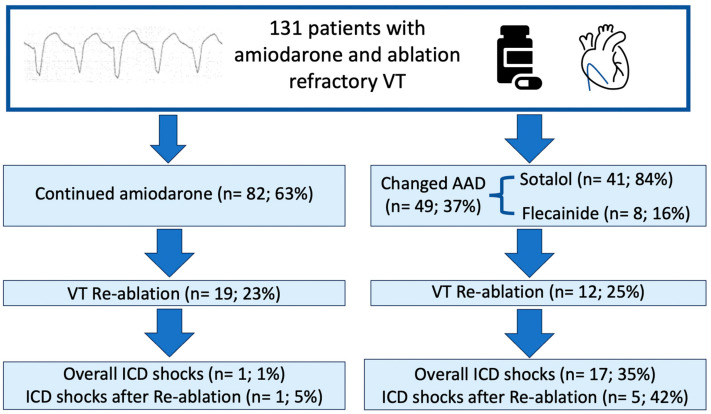
Incidence of appropriate ICD shocks after continued AAD therapy with amiodarone or changed AAD therapy.

During follow-up, a total of 31 (23.7%) patients underwent repeat VT ablation because of recurring VT (Figure 1). Among patients with continued amiodarone therapy but without VT ablation during follow-up (*n* = 63; 76.8% of patients with continued amiodarone), none experienced further ICD shocks. Among those who underwent VT re-ablation in the presence of continued chronic amiodarone therapy (*n* = 19; 23.2% of patients with continued amiodarone), one patient (5.3%) experienced an ICD shock during follow-up (Table 3; Figure 1).

Five flecainide patients and seven sotalol patients underwent VT re-ablation. In two and five of those, no further ICD shocks occurred during follow-up, whereas three and two patients presented with recurrent VT requiring ICD shocks, respectively. Most patients with continued amiodarone therapy who underwent VT re-ablation had an ischemic cardiomyopathy (52.6%).

**Figure 2:**
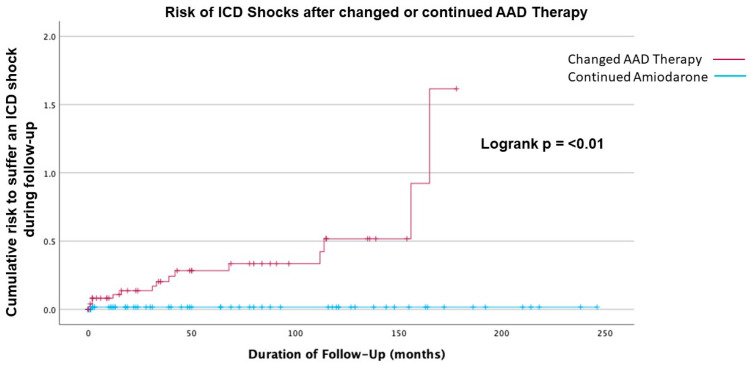
Kaplan–Meier analysis of risk for appropriate ICD shocks after either changed vs. continued amiodarone therapy.

There was a clear difference in the incidence of ICD shocks in patients undergoing a change from amiodarone to flecainide or sotalol (*p* < 0.01) compared to those staying on amiodarone (Figure 1). Also, patients with a non-ischemic cardiomyopathy experienced significantly more shocks than patients with an ischemic cardiomyopathy (*n* = 13 (21.0%) vs. *n* = 5 (7.2%), *p =* 0.02; Table 4). Changing AAD therapy was also significantly associated with a risk of suffering an ICD shock in multivariate regression analysis (OR 34.9, CI 95%, 4.3; 283.8, *p* ≤ 0.01) (Table 5 and Figure 2). Using a uniform follow-up period across all patient cohorts of 2.9 years, there was still a significant difference in appropriate shock incidence in patients undergoing an AAD change compared to remaining on amiodarone therapy (*n* = 10 vs. *n* = 0, respectively; *p* ≤ 0.01). However, applying these criteria, logistic regression could not derive a significantly increased risk of suffering an ICD shock after AAD therapy change (OR 1.75, CI 95%, 0.5; 6.4, *p* = 0.39).

## 6. Discussion

Amiodarone reduces arrhythmias in patients with cardiomyopathy-associated VT more effectively than other AADs [1,28]. Notably, the combination of guideline-directed medical therapy (GDMT) and guideline-directed device therapy (GDDT) has shown clear benefits, as evidenced, e.g., by a propensity score-adjusted subgroup analysis of the PARADIGM-HF trial [29]. In particular, the use of angiotensin receptor–neprilysin inhibitors appears to reduce the risk of sudden cardiac death (SCD), especially in ICD patients. This suggests a synergistic effect between ICD therapy and GDMT, with a 56% lower risk of SCD observed in patients receiving GDMT—an effect that was even more pronounced in those with an ICD compared to those without. In patients with heart failure, amiodarone reduced the risk of SCD with no effect on the overall mortality [30], as shown by, e.g., the AMIOVIRT study [31], which randomized 103 patients with non-ischemic cardiomyopathy, LVEF ≤ 35%, and asymptomatic non-sustained VT to an ICD or amiodarone. The study reported no significant difference in all-cause mortality during follow-up.

Due to its significant side effects, amiodarone therapy needs to be evaluated carefully and individually, and regular follow-up visits are mandatory. Sotalol as an alternative class III AAD has a higher proarrhythmic potential [17,32] whereas class I AADs are not considered as safe as amiodarone [33,34]. Based on the historic CAST trial [35], current guidelines [1] do not recommend treatment with class I AADs in patients with reduced ejection fraction or relevant structural heart disease due to suspected excess mortality and lack of data. Therefore, in patients with a reduced ejection fraction, class I AADs are usually only used as a bail-out therapy in the case of amiodarone, sotalol and/or catheter ablation [36,37], refractory VT, and recurrent ICD shocks [18,28].

Data on the effectiveness of changing or escalating AAD regimens due to the ineffectiveness or intolerance of chronic amiodarone treatment is scarce. The Ventricular Tachycardia Ablation versus Escalation of Antiarrhythmic drugs (“VANISH”) Trial [38] reported a rate of ICD shocks of almost 70% in 259 patients with an ischemic cardiomyopathy and significantly reduced LVEF (mean LVEF 31.2 ± 10.7%) during a mean follow-up of 27 ± 17 months after escalated antiarrhythmic therapy and therefore raised suspicion concerning the risk–benefit balance of drug escalation. In a sub-analysis of the trial [39], mexiletine in addition to chronic high-dose amiodarone therapy with ≥300 mg daily proved to have only a limited effect on VT recurrence. Our analysis of a daily practice cohort is in line with these results as we observed a significant difference in the incidence of ICD shocks of patients with changed as compared to continued amiodarone therapy in patients with a lower mean LVEF (27.7 ± 6.9%) compared to the VANISH trial (31.2 ± 10.7%). This is further consolidated by our multivariate regression analysis which revealed a significantly increased risk of further appropriate ICD shocks after changing AAD therapy. The increase in VT may not be directly related to the drug change but rather mirror a progressive cardiomyopathy with a more fertile arrhythmogenic substrate. This could explain the higher incidence of appropriate ICD shocks in patients who switched AAD therapy. Since our cohorts do not significantly differ in left ventricular ejection fraction, the impact of underlying cardiomyopathy seems less relevant, and the use of cardiac resynchronization therapy, which is associated with a reduction in ICD therapies as it improves ventricular function [40,41], was evenly distributed between groups. Patients with non-ischemic cardiomyopathy experienced significantly more shocks during follow-up than patients with an underlying ischemic cardiomyopathy (*p* = 0.02). This is most likely related to a more complex underlying of arrhythmic substrate in patients with non-ischemic cardiomyopathy [42]. Thus, the increase in appropriate ICD shocks may also reflect progression of the arrhythmogenic substrate rather than a direct effect of the AAD regimen.

## 7. Limitations

The underlying analysis has a retrospective design and uses non-randomized data analyses with an inherent selection bias of patients and therefore remains hypothesis generating. But, as baseline characteristics do not differ significantly between subgroups, comparability seems justified. Based on the present results, changes in chronic amiodarone therapy should be critically rethought, though we can only speculate on the ICD intervention rates if the patients had been left on amiodarone. Generalizability and clinical application may also be limited due to the sample size of our cohort and differences in follow-up. To address this limitation, we added a calculation using a uniform follow-up period across all cohorts. In addition, ATP/VT recurrences were not systematically registered. We cannot exclude that failure of amiodarone in those who were changed to other drugs was related to a change/progression of the underlying arrhythmogenic substrate, so that the increase in arrhythmias after changing AAD therapy may not be drug related but rather caused by the underlying substrate or other unknown influencing factors. As too few patients with a S-ICD were included in this study (*n* = 2), a valid subgroup analysis or comparison to TV-ICDs was not possible. Furthermore, due to the relatively small number of patients in the study, the options for statistical analysis were limited. A larger dataset, especially including more patients in the subgroup with changed AAD therapy, would have allowed a more sophisticated statistical analysis, e.g., a Cox proportional hazards regression.

## 8. Conclusions

In this investigation, we observed an increased incidence and risk of appropriate ICD shocks after changing the AAD regimen from chronic amiodarone to either sotalol or flecainide compared to continued amiodarone therapy in patients with structural heart disease, severely impaired LV function, and at least one previous VT ablation. Our results challenge the strategy of changing chronic amiodarone therapy in ICD patients. Further evidence is warranted to answer the question of what is the optimal AAD therapy for patients with recurrent VT and appropriate ICD shocks.

## Figures and Tables

**Table 1:** Baseline characteristics of patients with continued amiodarone or changed AAD therapy.

	Overall	AAD Therapy Changed	Amiodarone Continued	*p*-Value
*n*	131	49 (37.4)	82 (62.6)	<0.01
Shocks during follow-up (%)	18 (13.7)	17 (34.7)	1 (1.2)	<0.01
Death during follow-up (%)	11 (8.4)	7 (14.3)	4 (4.9)	0.06
Age (years, ±SD)	66.0 (12.8)	63.5 (11.8)	67.5 (13.2)	0.19
Sex (male, %)	108 (82.4)	44 (89.8)	64 (78.1)	0.09
Height (cm, ±SD)	174.3 (20.6)	177.7 (9.6)	172.8 (19.7)	0.06
Weight (kg, ±SD)	88.6 (18.8)	88.9 (19.0)	83.9 (15.4)	0.06
BMI (kg/m^2^, ±SD)	27.9 (5.4)	28.1 (5.3)	27.8 (5.5)	0.72
Hyperlipoproteinemia (%)	52 (39.7)	24 (49.0)	28 (34.2)	0.10
Nicotine abuse (%)	43 (32.8)	17 (34.7)	26 (31.7)	0.72
Arterial hypertension (%)	93 (71.0)	35 (71.4)	58 (70.7)	0.93
Myocardial infarction (%)	42 (32.1)	18 (36.7)	24 (29.3)	0.38
LVEF (%, ±SD)	27.7 (6.9)	27.5 (6.7)	27.8 (7.0)	0.86
Stroke (%)	9 (6.9)	5 (10.2)	4 (4.9)	0.25
CHA_2_DS_2_-VASc-Score (±SD)	2.9 (1.6)	3.0 (1.7)	2.7 (1.5)	0.93
Ischemic cardiomyopathy (%)	69 (52.7)	23 (46.9)	46 (56.1)	0.31
Non-ischemic cardiomyopathy (%)	62 (47.3)	26 (53.1)	36 (43.9)	0.31
History of SVT ablation (%)	2 (1.5)	2 (4.1)	0 (0.0)	0.07
History of AF (%)	54 (41.2)	19 (38.8)	35 (42.7)	0.66
VT ablation during FU (%)	31 (23.7)	12 (24.5)	19 (23.2)	0.87
Baseline Medication				
Betablocker (%)	111 (84.7)	39 (79.6)	72 (87.8)	0.21
Oral anticoagulation (%)	66 (50.4)	24 (49.0)	42 (51.2)	0.81
Phenprocoumon (%)	43 (65.2)	18 (75.0)	25 (59.5)	0.21
Apixaban (%)	11 (16.7)	3 (12.5)	8 (19.1)	0.49
Edoxaban (%)	4 (6.1)	0 (0.0)	4 (9.5)	0.12
Rivaroxaban (%)	7 (10.6)	3 (12.5)	4 (9.5)	0.71
Dabigatran (%)	1 (1.5)	0 (0.0)	1 (2.4)	0.45
Baseline Laboratory Results				
Potassium (mmol/L, ±SD)	4.4 (0.8)	4.2 (0.5)	4.4 (0.6)	0.02
Creatinine (mg/dL, ±SD)	1.4 (1.0)	1.2 (0.4)	1.3 (0.5)	0.16
ECG Characteristics				
Right bundle branch block (%)	13 (9.9)	5 (10.2)	8 (9.8)	0.94
Left bundle branch block (%)	38 (29.0)	12 (24.5)	26 (31.7)	0.38
QRS (ms, ±SD)	133.6 (32.7)	126.3 (30.9)	139.7 (33.1)	<0.01
QTc (ms, ±SD)	461.6 (57.8)	453.6 (70.3)	469.6 (41.2)	0.06
QT (ms, ±SD)	469.8 (47.5)	491.4 (44.5)	464.4 (47.4)	<0.01
Device Characteristics				
VVI-ICD (%)	63 (48.1)	28 (57.1)	35 (42.7)	0.11
DDD-ICD (%)	37 (28.2)	10 (20.4)	27 (32.9)	0.13
CRT-D (%)	29 (22.1)	11 (22.5)	18 (22.0)	0.95
S-ICD (%)	2 (1.5)	0 (0.0)	2 (2.4)	0.28
Device upgrade during follow-up (%)	43 (32.8)	15 (30.6)	28 (34.2)	0.67

Qualitative data are presented as *n* (%); quantitative data are presented as mean ± SD or median (IQR); BMI = body mass index; LVEF = left ventricular ejection fraction; ICD = implantable cardioverter defibrillator; VT = ventricular tachycardia; CRT-D = cardiac resynchronization therapy-defibrillator device; S-ICD = subcutaneous implantable defibrillator device; VKA = vitamin K receptor antagonist; SVT = supraventricular tachycardia; percentages are derived from the total numbers in each column.

**Table 2:** Baseline characteristics of patients with amiodarone- and ablation-treated VT in the presence of reduced LVEF ≤ 40% either with or without an ICD shock during follow-up.

	Overall	No Shock	Shock	*p*-Value
*n*	131	113 (86.3)	18 (13.7)	<0.01
Continued Amiodarone (%)	82 (62.6)	81 (71.7)	1 (5.6)	<0.01
Changed to Sotalol (%)	41 (31.3)	28 (24.8)	13 (72.2)	<0.01
Changed to Flecainide (%)	8 (6.1)	4 (3.5)	4 (22.2)	<0.01
Death during follow-up (%)	11 (8.4)	8 (7.1)	3 (16.7)	0.17
Age (years, ±SD)	66.0 (12.8)	66.7 (12.7)	61.8 (13.2)	0.13
Sex (male, %)	108 (82.4)	91 (80.5)	17 (94.4)	0.15
Height (cm, ±SD)	174.3 (20.6)	174.2 (17.4)	178.4 (7.9)	0.23
Weight (kg, ±SD)	88.6 (18.8)	86.0 (18.1)	86.8 (12.1)	1.00
BMI (kg/m^2^, ±SD)	27.9 (5.4)	28.0 (5.7)	27.4 (3.9)	0.41
Hyperlipoproteinemia (%)	52 (39.7)	44 (38.9)	8 (44.4)	0.65
Nicotine abuse (%)	43 (32.8)	37 (32.7)	6 (33.3)	0.92
Arterial hypertension (%)	93 (71.0)	81 (71.7)	12 (66.7)	0.66
Myocardial infarction (%)	42 (32.1)	34 (30.9)	8 (44.4)	0.26
LVEF (%, ±SD)	27.7 (6.9)	27.7 (7.0)	27.4 (6.3)	0.86
Stroke (%)	9 (6.9)	6 (5.3)	3 (16.7)	0.07
CHA_2_DS_2_-VASc-Score (±SD)	2.9 (1.6)	3.1 (1.7)	2.6 (1.5)	0.95
Ischemic cardiomyopathy (%)	69 (52.7)	62 (54.9)	7 (38.9)	0.21
Non-ischemic cardiomyopathy (%)	62 (47.3)	51 (45.1)	11 (61.1)	0.21
History of SVT ablation (%)	2 (1.5)	1 (0.9)	1 (5.6)	0.13
History of AF (%)	54 (41.2)	47 (41.6)	7 (38.9)	0.83
VT ablation during follow-up (%)	31 (23.7)	25 (22.1)	6 (33.3)	0.30
Baseline Medication				
Betablocker (%)	111 (84.7)	95 (84.1)	16 (88.9)	0.60
Oral anticoagulation (%)	66 (50.4)	57 (50.4)	9 (50.0)	0.97
Phenprocoumon (%)	43 (65.2)	35 (61.4)	8 (88.9)	0.11
Apixaban (%)	11 (16.7)	11 (19.3)	0 (0.0)	0.15
Edoxaban (%)	4 (6.1)	4 (7.0)	0 (0.0)	0.42
Rivaroxaban (%)	7 (10.6)	6 (10.5)	1 (11.1)	0.96
Dabigatran (%)	1 (1.5)	1 (1.8)	0 (0.0)	0.69
Baseline Laboratory Results				
Potassium (mmol/L, ±SD)	4.4 (0.8)	4.4 (0.5)	4.3 (0.6)	0.35
Creatinine (mg/dL, ±SD)	1.4 (1.0)	1.2 (0.4)	2.1 (0.5)	<0.01
ECG Characteristics				
Right bundle branch block (%)	13 (9.9)	10 (8.9)	3 (16.7)	0.31
Left bundle branch block (%)	38 (29.0)	31 (27.4)	7 (38.9)	0.32
QRS (ms, ±SD)	133.6 (32.7)	133.9 (33.3)	123.5 (32.4)	0.94
QTc (ms, ±SD)	461.6 (57.8)	466.0 (41.6)	438.3 (70.9)	<0.01
QT (ms, ±SD)	469.8 (47.5)	462.7 (45.5)	442.5 (61.0)	0.05
Device Characteristics				
VVI-ICD (%)	63 (48.1)	53 (46.9)	10 (55.6)	0.49
DDD-ICD (%)	37 (28.2)	32 (28.3)	5 (27.8)	0.97
CRT-D (%)	29 (22.1)	26 (23.0)	3 (16.7)	0.55
S-ICD (%)	2 (1.5)	2 (1.8)	0 (0.0)	0.57
Device upgrade (%)	43 (32.8)	35 (31.0)	8 (44.4)	0.26
Change in antiarrhythmic therapy from amiodarone to sotalol or flecainide during follow-up				
Change in antiarrhythmic drug	49 (37.4)	32 (28.3)	17 (94.4)	<0.01
Ineffective (%)	20 (40.8)	11 (34.4)	13 (76.5)	<0.01
Not tolerated (%)	8 (16.3)	8 (25.0)	2 (11.8)	0.22
Other *	21 (42.9)	13 (40.6)	2 (11.8)	0.02

Qualitative data are presented as *n* (%); quantitative data are presented as mean ± SD or median (IQR); BMI = body mass index; LVEF = left ventricular ejection fraction; ICD = implantable cardioverter defibrillator; VT = ventricular tachycardia; CRT-D = cardiac resynchronization therapy-defibrillator device; S-ICD = subcutaneous implantable defibrillator device; SVT = supraventricular tachycardia; percentages are derived from the total numbers in each column. * Including patient wish, incompliance, and unknown reason.

**Table 3:** Baseline characteristics of patients with continued amiodarone with or without VT re-ablation during follow-up.

	Overall	VT Ablation	No VT Ablation	*p*-Value
*n*	82 (62.6)	19 (23.2)	63 (76.8)	<0.01
Shocks during follow-up (%)	1 (1.2)	1 (5.3)	0 (0.0)	0.07
Death during follow-up (%)	4 (4.9)	1 (5.3)	3 (4.8)	0.93
Age (years, ±SD)	67.5 (13.2)	67.4 (11.7)	67.5 (13.7)	0.97
Sex (male, %)	64 (78.1)	17 (89.5)	47 (74.6)	0.03
Height (cm, ±SD)	172.8 (19.7)	174.9 (5.0)	170.6 (19.0)	0.05
Weight (kg, ±SD)	83.9 (15.4)	84.9 (14.7)	82.7 (16.2)	0.56
BMI (kg/m^2^, ±SD)	27.8 (5.5)	27.8 (4.9)	27.3 (6.1)	0.70
Hyperlipoproteinemia (%)	28 (34.2)	5 (26.3)	23 (36.5)	0.41
Nicotine abuse (%)	26 (31.7)	6 (31.6)	20 (31.8)	0.98
Arterial hypertension (%)	58 (70.7)	12 (63.2)	46 (73.0)	0.41
Myocardial infarction (%)	24 (29.3)	6 (31.6)	18 (28.6)	0.80
LVEF (%, ±SD)	27.8 (7.0)	29.0 (7.7)	27.4 (6.8)	0.38
Stroke (%)	4 (4.9)	3 (15.8)	1 (1.6)	0.01
CHA_2_DS_2_-VASc-Score (points, ±SD)	2.7 (1.5)	3.3 (1.4)	3.4 (1.5)	0.78
Ischemic cardiomyopathy (%)	46 (56.1)	10 (52.6)	36 (57.1)	0.73
Non-ischemic cardiomyopathy (%)	36 (43.9)	9 (47.4)	27 (42.9)	0.73
History of SVT ablation (%)	0 (0.0)	0 (0.0)	0 (0.0)	NA
History of AF (%)	35 (42.7)	4 (21.1)	31 (49.2)	0.83
Baseline Medication				
Betablocker (%)	72 (87.8)	17 (89.5)	55 (87.3)	0.80
Oral anticoagulation (%)	42 (51.2)	4 (21.1)	38 (60.3)	<0.01
Phenprocoumon (%)	25 (59.5)	1 (25.0)	24 (63.2)	0.14
Apixaban (%)	8 (19.1)	1 (25.0)	7 (18.4)	0.75
Edoxaban (%)	4 (9.5)	2 (50.0)	2 (5.3)	<0.01
Rivaroxaban (%)	4 (9.5)	0 (0.0)	4 (10.5)	0.50
Dabigatran (%)	1 (2.4)	0 (0.0)	1 (2.6)	0.75
Baseline Laboratory Results				
Potassium (mmol/L, ±SD)	4.4 (0.6)	4.5 (0.6)	4.5 (0.7)	1.00
Creatinine (mg/dL, ±SD)	1.3 (0.5)	1.4 (0.4)	1.4 (0.9)	1.00
ECG Characteristics				
Right bundle branch block (%)	8 (9.8)	1 (5.3)	7 (11.1)	0.46
Left bundle branch block (%)	26 (31.7)	6 (31.6)	20 (31.8)	0.99
QRS (ms, ±SD)	139.7 (33.1)	142.3 (41.9)	154.0 (34.3)	0.22
QTc (ms, ±SD)	469.6 (41.2)	471.1 (49.5)	477.0 (44.4)	0.62
QT (ms, ±SD)	464.4 (47.4)	436.7 (15.3)	468.4 (50.9)	<0.01
Device Characteristics				
VVI-ICD (%)	35 (42.7)	8 (42.1)	27 (42.9)	0.95
DDD-ICD (%)	27 (32.9)	6 (31.6)	21 (33.3)	0.89
CRT-D (%)	18 (22.0)	4 (21.1)	14 (22.2)	0.92
S-ICD (%)	2 (2.4)	1 (5.3)	1 (1.6)	0.36
Device upgrade during follow-up (%)	28 (34.2)	3 (15.8)	25 (39.7)	0.06

Qualitative data are presented as *n* (%); Quantitative data are presented as mean ± SD or median (IQR); BMI = body mass index; LVEF = left ventricular ejection fraction; ICD = implantable cardioverter defibrillator; VT = ventricular tachycardia; CRT-D = cardiac resynchronization therapy-defibrillator device; S-ICD = subcutaneous implantable defibrillator device; SVT = supraventricular tachycardia; percentages are derived from the total numbers in each column.

**Table 4:** Baseline characteristics of patients with either an ischemic or non-ischemic cardiomyopathy.

	Overall	ICM	NICM	*p*-Value
*n*	131	69 (52.7)	62 (47.3)	
Shocks during follow-up (%)	18 (13.7)	5 (7.2)	13 (21.0)	0.02
Death during follow-up (%)	11 (8.4)	7 (10.1)	4 (6.5)	0.46
Age (years, ±SD)	66.0 (12.8)	64.4 (13.9)	62.8 (15.4)	0.53
Sex (male, %)	108 (82.4)	54 (78.3)	54 (87.1)	0.97
BMI (kg/m^2^, ±SD)	27.9 (5.4)	28.1 (5.4)	28.5 (6.2)	0.69
Hyperlipoproteinemia (%)	52 (39.7)	28 (40.6)	24 (38.7)	0.82
Nicotine abuse (%)	43 (32.8)	22 (31.9)	21 (33.9)	0.81
Arterial hypertension (%)	93 (71.0)	46 (66.7)	47 (75.8)	0.25
Myocardial infarction (%)	42 (32.1)	27 (39.1)	15 (24.2)	0.33
LVEF (%, ±SD)	27.7 (6.9)	35.9 (12.0)	35.0 (11.2)	0.66
Stroke (%)	9 (6.9)	5 (7.2)	4 (6.5)	0.87
CHA_2_DS_2_-VASc-Score (±SD)	2.9 (1.6)	3.2 (1.4)	3.0 (1.5)	0.43
Baseline Medication				
Betablocker (%)	111 (84.7)	56 (82.4)	55 (88.7)	0.31
Oral anticoagulation (%)	66 (50.4)	21 (30.9)	45 (72.6)	<0.01
ECG Characteristics				
Right bundle branch block (%)	13 (9.9)	8 (12.7)	5 (8.1)	0.39
Left bundle branch block (%)	38 (29.0)	11 (17.5)	27 (43.6)	<0.01
QRS (ms, ±SD)	133.6 (32.7)	133.3 (33.3)	143.2 (32.4)	0.09
QTc (ms, ±SD)	461.6 (57.8)	468.5 (48.1)	460.8 (76.4)	0.49
QT (ms, ±SD)	469.8 (47.5)	452.1 (42.5)	482.1 (48.0)	<0.01
Device Characteristics				
VVI-ICD (%)	63 (48.1)	35 (50.7)	28 (45.2)	0.53
DDD-ICD (%)	37 (28.2)	19 (27.5)	18 (29.0)	0.85
CRT-D (%)	29 (22.1)	8 (11.6)	21 (33.9)	<0.01
S-ICD (%)	2 (1.5)	2 (2.9)	0 (0.0)	0.18

Qualitative data are presented as *n* (%); quantitative data are presented as mean ± SD or median (IQR); NICM = non-ischemic cardiomyopathy; ICM = ischemic cardiomyopathy; BMI = body mass index; LVEF = left ventricular ejection fraction; ICD = implantable cardioverter defibrillator; VT = ventricular tachycardia; CRT-D = cardiac resynchronization therapy-defibrillator device; S-ICD = subcutaneous implantable defibrillator device; percentages are derived from the total numbers in each column.

**Table 5:** Multivariate logistic regression analysis regarding the risk of suffering an appropriate ICD shock with either continued amiodarone or changed AAD therapy.

Logistic Regression						
Characteristic	Univariable			Multivariable		
	OR	95% CI	*p*-Value	OR	95% CI	*p*-Value
Change in antiarrhythmic therapy	24.4	5.5; 107.8	<0.01	34.9	4.3; 283.8	<0.01
Age (years)	0.98	0.90; 1.06	0.67			
Sex	1.96	0.61; 33.3	0.63			
BMI (kg/m^2^)	1.01	0.86; 1.19	0.86			
LVEF (%)	0.99	0.90; 1.08	0.88			
CHA_2_DS_2_-VASc-Score (pts.)	0.95	0.48; 1.87	0.87			
Structural heart disease	1.62	0.26; 10.0	0.60			
QRS width (ms)	1.01	0.98; 1.04	0.46			
QTc (ms)	1.00	0.98; 1.02	0.98			
Potassium (mmol/L)	1.05	0.88; 1.25	0.53			

OR = Odds Ratio; CI = confidence interval; BMI = body mass index; LVEF = left ventricular ejection fraction.

## Data Availability

Data available on request.
